# Carbolic Acid Used in Full Strength in Surgery

**Published:** 1894-01

**Authors:** Oscar H. Allis

**Affiliations:** Philadelphia, Pa.; Surgeon to the Presbyterian Hospital


### CARBOLIC ACID USED IN FULL STRENGTH IN SURGERY.

BY OSCAR H. ALLIS, M. D., PHILADELPHIA, PA.
Surgeon to the Presbyterian Hospital.

Surgeons in early days of antiseptic surgery attributed their success to carbolic acid. As introduced, it was employed in a dilute aqueous or oleaginous solution. For a time it was the sole antiseptic. To-day it is mainly used in general surgery as a bath for surgical instruments. Few surgeons will demand a reason for its abandonment. Few have not personally experienced its benumbing effects, and have thus been able to assign the collapse following its employment to something different than lost of blood, shock of operation or anæsthetic.

With such an experience of carbolic acid in its dilute form, I confess that I was quite astonished to learn from my friend, Dr. B. F. Gardner, of Bloomsburg, that he was in the habit of using the article in its full strength upon extensive cut surfaces, and that, too, with the happiest results. As this article owes its entire value to Dr. Gardner, I will give in detail his method.

When Lister introduced his paste, Dr. Gardner used it quite extensively. After an application to quite an extensive wound surface he was surprised to find it turn white, and that he had used pure carbolic acid. He therefore immediately washed the surface and dressed the wound, keeping it open until oozing had ceased. The case did so well that it inaugurated with him a line of treatment that he has extensively employed. As a typical application let me take an amputation of the female breast. After its removal and the ligation of the bleeding vessels carbolic acid crystals, dissolved in sufficient water for solution, are applied with a sponge to all parts of the cut surface. Immediately upon the application of the acid the tissues turn white, which is a guarantee of its thorough action. The wound surface is then washed with water previously sterilized by boiling and then approximated with provisions for drainage.

This is especially necessary, as for twenty-four hours the oozing must find ready exit. During the first few days there is a slight local hyperemia along the borders of approximation, but this declines without crisis.

Dr. Gardner claims for carbolic acid applied in officinal strength :

1. That no systemic absorption attends its use, and hence no danger, no shock.

2. That it is a local anæsthetic. Hence there is not as much pain after the operation.

3. That it is in a measure a hæmostatic, acting especially upon the capillary vessels.

I have taken the removal of the mamma only as an illustrative case. In all operations outside of the pleuritic and abdominal cavities, such as amputations and resections, Dr. Gardner resorts to it.

In hydrocele he lays open the sac freely, then applies carbolic acid to the tunica vaginalis, and concludes with packing or drainage. The operation is not followed by excess of any kind, and recovery is prompt. He has used it in gunshot wounds of the knee and ankle. If he gets such a case after suppuration has set in he freely opens the joint, applies the carbolic acid to every part, washes out all excess freely, secures ample drainage with fixation, and confidently awaits the result. Anchylosis may follow, but this will depend on the extent of the injury, the delay in treatment, the conduct of the patient. Dr. Gardner has used bichloride of mercury, hydrogen peroxide, iodoform, etc ; none of them has answered the claims made for them ; all have disappointed him, but pure carbolic acid *never.*

I have said that Dr. Gardner does not use this upon serous membranes, *i. e.*, within the abdomen. I must modify this statement. In a case of strangulated hernia, in which he found patches of sphacelus—not deep, but threatening—he cautiously applied the pure acid and returned the gut. Fortunately, the strangulation had been arrested by operation in time to save the gut. Nothing eventful in the subsequent history, which was speedy.

I do not know Dr. Gardner's theory of the actions of this powerful drug, and shall attempt no explanation. The turning of the wound surface white is due probably to the coagulation of the albumin of the tissues and fluids of the wound surface, and not that the acid has a necrotic effect. That it does not produce a true destruction of tissue may be inferred that after a large breast or thigh amputation he will have primary union and no suppuration. In its use in hydrocele a half drachm or more is injected into the tunica vaginalis, and resolution without suppuration ensues. It is possible that by its action upon the wound surface an action similar to that obtained by heat may be produced, and thus facilitate repair.

I will conclude this article by briefly stating my own experience with it.

On entering the wards of the Presbyterian Hospital I found that one of my amputations of the thigh had not done well, and looking at the stump found it swoolen and of an angry threatening character. The seam of approximation was perfect. I therefore removed all the sutures, and separating the flaps found them almost in a stage of gangrene. Taking carbolic acid pure, I applied it freely, pressing it into the tissues with the sponge applicator, removed the excess, and, packing the space between the flaps renewed the dressing. This was done without anæsthetic and without apparent pain. The exposed surfaces soon began to granulate, when they were approximated and recovery soon followed. I have also frequently applied it upon a carrier with cotton to sinuses and after curetting glands.

